# The rising importance of *Triatoma rubrofasciata*

**DOI:** 10.1590/0074-02760140446

**Published:** 2015-05

**Authors:** Jean-Pierre Dujardin, Truong Xuan Lam, Pham Thi Khoa, Christopher John Schofield

**Affiliations:** 1 Unité Mixte de Recherche 177-Interactions Hôte-Vecteur-Parasite-Enrironnement dans les Maladies Tropicales Négligées dues aux Trypanosomatidés, Centre International de Recherches Agronomiques pour le Développement, Institut de Recherches pour le Développement, Campus international de Baillarguet, Montpellier, France; 2 Institute of Ecology and Biological Resources, Vietnam Academy of Sciences and Technology, Hanoi, Vietnam; 3 National Institute of Malariology, Parasitology and Entomology, Hanoi, Vietnam; 4 London School of Tropical Medicine and Hygiene, London, UK

**Keywords:** Vietnam, Triatoma rubrofasciata, Trypanosoma sp

## Abstract

The migration of invasive vector species has contributed to the worldwide extension of infectious diseases such as dengue (Aedes aegypti) and chikungunya (Aedes albopictus). It is probably a similar behaviour for certain vectors of Chagas disease which allowed it to become a continental burden in Latin America. One of them, Triatoma rubrofasciata has also been spreading throughout the tropical and subtropical world. Here, the recent and massive peridomestic presence of T. rubrofasciata in Vietnam cities is reported, and tentatively explained, highlighting the need for improved entomological surveillance.

In the XVIII century, Bernardin de Saint-Pierre described, at the island of La Reunion, a kind of *punaise des bois* ("sylvatic stinkbug") by these words: "... its bite is more dangerous than the scorpion; it is followed by a tumour the size of a pigeon's egg, which dissipates after five or six days" [translated from Martin (1820)]. Three centuries later, we received from the same La Reunion island a few pictures of a bug "found in the bed of a child", a bug we identified as *Triatoma rubrofasciata* (Hemiptera: Reduviidae). This simple story sent us a kind of warning because, after three centuries, the bug was no more a *punaise des bois*, it had come to be a bug "found in the bed of a child".

The domestic habits of some haematophagous Hemiptera of the subfamily Triatominae represent the main risk for humans to be infected by *Trypanosoma cruzi* - causative agent of Chagas disease. Since these insects are found predominantly in the New World, particularly in Latin America, Chagas disease is an endemic disease typically considered as limited to the American continent. However, a number of people infected by *T. cruzi* acquired the infection in the Old World through blood transfusion from Latin immigrants unaware of their own infection [eg., Gascon et al. (2010)]. Since the parasite can be transmitted by blood transfusion and organ transplant, the increasing transcontinental exchanges mean that Chagas disease is no longer restricted to the American continent (Coura & Viñas 2010, Tanowitz et al. 2011).

Migration of the insect vectors might also help to spread the disease beyond its previous limits. This could occur through the accidental transportation of infected bugs able to survive in other tropical and subtropical countries or of uninfected bugs that could become established and then catch the parasite from a Latin immigrant (Schofield et al. 2009 ). At present, the risk for such a scenario seems to be low, but it is supported by the situation recently denounced in Vietnam, where *T. rubrofasciata* develops a propensity to feed on humans. This short note presents some statistics about the peridomestic infestation by *T. rubrofasciata *in Vietnam and discusses the possible scenarios underlying this relatively recent phenomenon.

*T. rubrofasciata in Vietnam* - Although recorded in Vietnam since Kalshoven (1970) and recently confirmed in Vietnam by national entomologists (Truong 2004), it is only a few years ago that the tropicopolitan *T. rubrofasciata* became a cause of concern for health authorities. In the last decade, many people made complaints of being attacked by an "assassin" bug in different places of Vietnam - mainly Hanoi and other large cities - leading local health authorities to seek national and international entomological expertise. The incriminated bug was *T. rubrofasciata*. A national survey between 2010-2012 confirmed that it could be found in at least 21 provinces of Vietnam, with a seasonal peak of adult bugs found from June-September. Heavy infestations, involving hundreds of insects per house, were reported both from urban and rural areas, with the highest densities found in urban areas (Truong & Dujardin 2013, Van Chau et al. 2013). Major cities were particularly affected, especially Hanoi (Fig. 1) which is located at more than 100 km from the coast at Hai Phong. The common micro-habitat was found close to human dwellings where wood, firewood or waste were accumulated. In the Long Bien and Tu Liem districts of Hanoi, *T. rubrofasciata* populations were concentrated in large peridomestic shelters with all development stages (eggs, nymphs and adults) readily observed. In general, these shelters were also infested with rats, but the bugs were also found in association with chickens (Truong & Dujardin 2013). In big cities such as Hanoi, Danang and Ho Chi Minh, adult specimens of *T. rubrofasciata* were also reported in buildings from the ground floor up to the eighth floor, biting sleeping people and hiding in their beds.

**Fig. 1:**
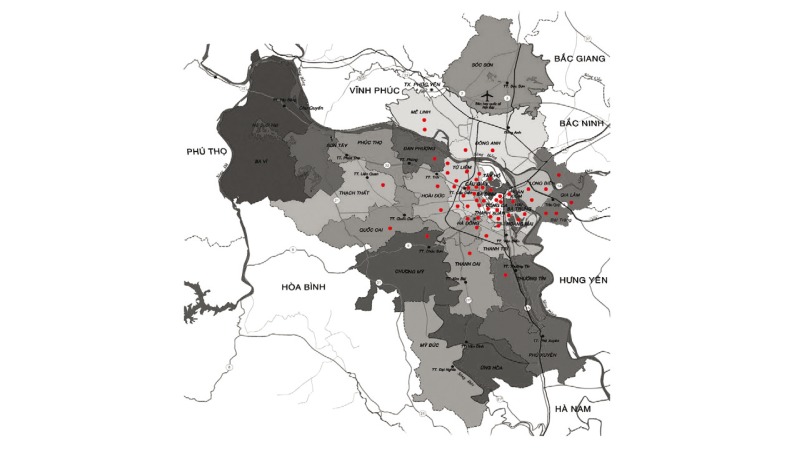
map of Hanoi Province. Red dots correspond to sites positive for Triatoma rubrofasciata (Truong & Dujardin 2013).

Rural infestations were apparently less dense, but ge- ographically extended deep into the continent. The peri- domestic presence of *T. rubrofasciata* was also reported to the northeast of Hanoi, extending across the frontier well into southern China (W Cai, unpublished observations).

*T. rubrofasciata* is known to transmit at least two parasites: *T. cruzi* in Latin America and *Trypanosoma conorhini *worldwide*. *Excluding infections in Latin American migrants, *T. cruzi* is found exclusively in the New World and is infective to humans and most other mammals. By contrast, *T. conorhini *was first recorded in the intestine of *T. rubrofasciata* in Madras, in 1909, and subsequently reported from *T. rubrofasciata* in many other locations around the world, including Latin America (Miles 2013). A *T. conorhini*-like trypanosome, indistinguishable from *T. conorhini *by random amplification of polymorphic DNA was discovered recently in another species of triatomine bug, *Linshcosteus* *karupus* in India (Miles 2013). In the recent surveys in Vietnam, trypanosomatid forms have been recognised in the intestine of the bugs at a high frequency (50-90%); the parasite is not definitively identified, although its general morphology indicates a *Megatrypanum *species such as *T. conorhini*.

The natural host of *T. conorhini *was discovered in 1937 in Java: rats of the species *Rattus rattus* L. (the ship rat) were shown to be infected by feeding uninfected bugs on them (xenodiagnosis). Rats were also shown to eat infected bugs when given the opportunity and to become infected. Laboratory experiments on mice suggest that the natural route of the rat infection is by contamination with bug faeces and not by the bite of the infected bug (Miles 2013).

It is not known if humans have ever been infected with *T. conorhini*. So far in Vietnam, no trypanosome parasite has been detected in Giemsa-stained blood smears from blood taken seven-10 days after being bitten by *T. rubrofasciata* (Van Chau et al. 2013), but contamination of food with triatomine bug faeces might occur, leading to exposure to infection by the oral route. Another epidemiological consideration is the spread of human immunodeficiency virus infection, which might lead to the occurrence of *T. conorhini* infections in immunocompromised patients (Miles 2013).

Today, *T. rubrofasciata* in Vietnam is a serious nuisance. People bitten by the bug (mostly on legs and arms) presented swelling and itching at the bite (Fig. 2), sometimes with consecutive local skin infection. In Hanoi, 24% of cases also involved severe fever lasting one-two days (Truong & Dujardin 2013), although for the whole of Vietnam the average fever rate following a bite was around 5% (Van Chau et al. 2013). Repeated exposure to triatomine bites or to triatomine faeces may cause immunological reactions (Walter et al. 2012) and sometimes severe anaphylaxis that can be fatal (Teo & Cheah 1973, Lo Vecchio & Tran 2004, Wang & Peng 2006).

**Fig. 2:**
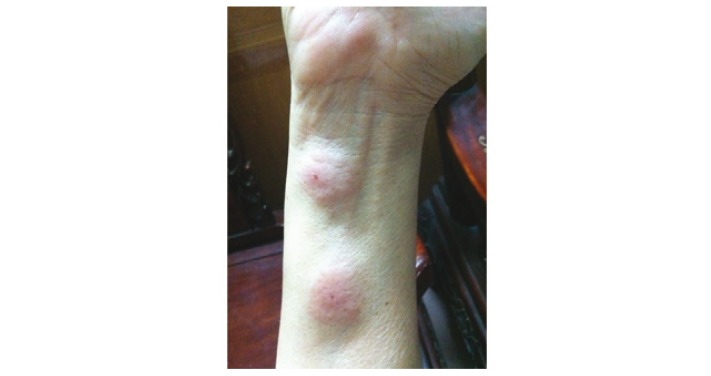
local skin reaction to the bite of Triatoma rubrofasciata (photo by one the authors, TXL).

*The origins of T. rubrofasciata in Vietnam* - Discussion of the origins of *T. rubrofasciata* in Vietnam, or elsewhere in the Old World, involves discussing the whole geographic distribution of Triatominae. The vast majority of species (95%) is limited to the New World, which suggests an American origin for the members of this subfamily. Outside the American continent, there are a few other species classified as belonging to the genus *Triatoma*, called the "Asiatic *Triatoma*", as well as a few species of another genus, the morphologically distinct *Linshcosteus* genus (Table I). Except for *T. rubrofasciata,* the *Triatoma* species described today in Asia do not occur in the New World and *vice versa*. Two hypotheses competed during decades, one defending an Asian origin for this species, the other suggesting an American origin (Table II).

**TABLE I.** Asiatic Triatominae

*Triatoma rubrofasciata*	Africa: Angola, Congo (Katanga), Guinea (Conakry), Saudi Arabia, Sierra Leone, South Africa, Tanzania Atlantic Ocean: Azores Asia -Pacific: Andaman Islands, Burma (Myanmar), Cambodia, Carolina Islands, China, Comoros Islands, India, Indonesia, Japan, Madagascar, Mauritius, Philippines, Reunion, Rodriguez Islands, Sri Lanka, Singapore, Seychelles, Taiwan, Thailand, Tonga, Vietnam Americas: Argentina (Buenos Aires), Brazil (Atlantic coastal areas), Cuba (and most other Caribbean islands), French Guiana, Suriname, Venezuela, United States of America (Florida, Hawaii)
*Triatoma amicitiae*	Sri Lanka
*Triatoma bouvieri*	Philippines, Vietnam
*Triatoma cavernicola*	Malaysia
*Triatoma leopoldi*	Australia (North Queensland), Indonesia
*Triatoma pugasi*	Indonesia (Java)
*Triatoma migrans*	India, Indonesia, Malaysia, Philippines, Thailand
*Triatoma sinica*	China
*Linshcosteus *sp.	India

the reported geographical distribution of T. rubrofasciata and other Old World Triatominae [updated from Ryckman and Archbold (1981) and Schofield et al. (2009)]. Note that many of the records are old (pre-1945) and may not reflect current distribution.

**TABLE II.** The possible origin of Triatoma rubrofasciata

An Asian origin for *T. rubrofasciata*	An American origin for *T. rubrofasciata*
Parasite	argument
Ryckman and Archbold (1981) noted that although *T. rubrofasciata* is the host of *Trypanosoma cruzi* and *Trypanosoma conorhini* in the New World, it had never met with *T. cruzi* in the Old World. This argument convinced them that *T. rubrofasciata* arrived in the New World with its natural parasite, *T. conorhini*, and was secondarily infected by *T. cruzi.*	The probability of being infected by *T. cruzi* a few centuries ago was not necessarily the same as today. The geographical expansion of *T. cruzi *to domestic animals (like *Rattus rattus*) in the Americas was the result of massive migration of domestic vectors like *Triatoma infestans*, *Rhodnius prolixus* or *Triatoma dimidiata*, that occurred only very recently (Schofield 1988, Dujardin 1998).
It has been suggested that the Asian monkey *Macaca* was the natural host of *T. conorhini *(Deane et al. 1986, Miles 2013).	It is not unlikely that the *Macaca* reared in a South American laboratory could have been infected during their transportation to other continents.
Insect	argument
There are other *Triatoma* species in Asia, not only *rubrofasciata* (Table I).	*T. rubrofasciata* would be the common ancestor of the other Asiatic species, including *Linshcosteus *(Hypsa et al. 2002).
The first collections of *T. rubrofasciata* were Asian. In fact, the species described by De Geer 1773 was collected *aux Indes* (believed to indicate the then Dutch East Indies or Indonesia). It represents the type species of the genus *Triatoma*.	Morphometric and molecular studies (Gorla et al. 1997, Patterson et al. 2001, Hypsa et al. 2002, Hwang & Weirauch 2012) suggest a New World origin for the “Asiatic clade” of Triatominae.

The hypothesis which is retained today is that *T. rubrofasciata*, because of its close association with domestic rats (especially *R. rattus*), has been transported on ships along early international trade routes during the XVI to XIX centuries (Schofield 1988, Gorla et al. 1997, Patterson et al. 2001). In this hypothesis of a New World origin however, more knowledge is needed to fully understand the distribution of both *T. cruzi* and *T. conorhini* (Table II). More data are also needed on *T. rubrofasciata*, since wild foci that might represent the original populations have never been reported.

In the hypothesis of *T. rubrofasciata* being an introduced species to Vietnam, the situation would correspond to the one already described in the New World, where domestic populations are frequently "exported" populations, i.e., bugs introduced with humans by passive transportation. As an insect introduced by humans outside its previous limits, *T. rubrofasciata* stayed with humans and adapted more and more to them.

The known domestic hosts of *T. rubrofasciata* are the rat - probably *Rattus exulans *and *Rattus norvegicus* in Vietnam (Morand 2013) - and the hen (Sherwin & Wood 1946). These two hosts support two different assumptions regarding its origins. The ship rat is the host that suits the idea of migration by sea, as it is readily found in the vessels carrying food, especially in past centuries where hygiene was less strict. Chickens however are the hosts that best fit the hypothesis of domestication. They are readily fed upon by most kissing bugs. In the rural areas of Latin America, they often appear as an important intermediate in the bugs' transition from silvatic to domestic ecotopes. Thinking of long voyages, one might imagine that for *T. rubrofasciata* the gate of "Departures" is the rat and the gate of "Arrivals" is the hen house (Dujardin 2013).

Whatever the hypothesis about *T. rubrofasciata* origins, the recent character of its massive and widespread infestation in Vietnam is unclear. An external trigger event could have been the fight against avian influenza. In the previous decade, campaigns against avian influenza (mainly H5N1) in Vietnam led to large-scale slaughter of peridomestic chickens - especially in urban and periurban areas. So it may be that this sudden loss of a primary host for the peridomestic *T. rubrofasciata* populations, triggered a host switch to available humans (Schofield et al. 2014).

*T. rubrofasciata* is an invasive species which, despite its large size and its irritating saliva, seems to be increasing its association with humans. In Vietnam it is already a matter of concern because of its bites and the bite reaction that can ensue. With increased migration of human populations and further development of anthropophilia of *T. rubrofasciata*, it seems entirely possible that *T. cruzi* infections reaching Asia could be picked up by urban *T. rubrofasciata.*

Because of its close association with synanthropic animals and humans, *T. rubrofasciata* could also spread throughout the tropical and subtropical world - even into some temperate areas (Gorla et al. 1997, Schofield et al. 2009). With increasing intercontinental transport, there is a risk of similar problems arising from the spread of other established domestic vectors of *T. cruzi *in South America: the danger of emerging trypanosomiasis would then be further increased. These arguments justify not only the vigilance, but also the prosecution of vector control in Latin America, the cradle of the most dangerous vectors.
